# The neurophenomenology of basic self-disturbance in early psychosis: Association with clinical outcome in an ultra-high risk sample

**DOI:** 10.1177/10398562251346619

**Published:** 2025-06-04

**Authors:** Vera A Barata, Suzie Lavoie, Łukasz Gawęda, Emily Li, Louis A Sass, Danny Koren, Patrick D McGorry, Bradley N Jack, Josef Parnas, Andrea Polari, Kelly Allott, Jessica A Hartmann, Marija Krcmar, Andreas R Rasmussen, Thomas J Whitford, Cassandra MJ Wannan, Barnaby Nelson

**Affiliations:** Department of Psychiatry, 26704 Hospital Professor Doutor Fernando Fonseca, Amadora, Portugal; Centre for Youth Mental Health, 590696 The University of Melbourne, Australia; Orygen, Parkville, VIC, Australia; Experimental Psychopathology Lab., Institute of Psychology, 111452 Polish Academy of Sciences, Warsaw, Poland; Centre for Youth Mental Health, 590696 The University of Melbourne, Australia; Orygen, Parkville, VIC, Australia; 5970 Rutgers University, New York, NY, USA; Psychology Department, 158199 University of Haifa, Haifa, Israel; Centre for Youth Mental Health, 590696 The University of Melbourne, Australia; Orygen, Parkville, VIC, Australia; Research School of Psychology, 188651 The Australian National University, Canberra, ACT, Australia; Department of Clinical Medicine, Faculty of Health and Medical Sciences, 652984 University of Copenhagen, Copenhagen, Denmark; Centre for Youth Mental Health, 590696 The University of Melbourne, Australia; Orygen, Parkville, VIC, Australia; Orygen Specialist Program, Melbourne, VIC, Australia; Centre for Youth Mental Health, 590696 The University of Melbourne, Parkville, VIC, Australia; Orygen, Parkville, VIC, Australia; Centre for Youth Mental Health, 590696 The University of Melbourne, Australia; Orygen, Parkville, VIC, Australia; Department of Public Mental Health, Medical Faculty Mannheim, Central Institute of Mental Health, Heidelberg University, Heidelberg, Germany; Centre for Youth Mental Health, 590696 The University of Melbourne, Parkville, VIC, Australia; Orygen, Parkville, VIC, Australia; Centre for Youth Mental Health, 590696 The University of Melbourne, Parkville, VIC, Australia; Department of Clinical Medicine, Faculty of Health and Medical Sciences, 652984 University of Copenhagen, Copenhagen, Denmark; Mental Health Center Amager, Copenhagen University Hospital, Copenhagen, Denmark; School of Psychology, 441985 University of New South Wales, Sydney, Australia; Centre for Youth Mental Health, 590696 The University of Melbourne, Parkville, VIC, Australia; Orygen, Parkville, VIC, Australia

**Keywords:** prodrome, psychosis, schizophrenia, phenomenology, neurocognition

## Abstract

**Introduction:**

We previously proposed a neurophenomenological model of schizophrenia, linking basic self-disturbance with neural deficits of source monitoring and aberrant salience. Baseline comparisons in ultra-high risk (UHR) and first-episode psychosis (FEP) samples indicated a relationship between basic self-disturbance and source monitoring deficits, but not aberrant salience. The current paper reports on the 12-month follow-up results in the UHR group (*n* = 43), focusing on the association between baseline variables and clinical outcomes.

**Methods:**

One-way ANOVA compared UHR-remitters (*n* = 18), UHR-persistent/transitioned to psychosis cases (*n* = 25) and FEP (*n* = 38) groups on baseline clinical and neuro-measures. Logistic regression assessed the baseline variables’ predictive power for UHR outcomes.

**Results:**

Higher baseline self-disturbance scores (EASE total) were found in the UHR persistence/transition and FEP groups compared to the UHR-remission group, and predicted worse UHR clinical outcomes. Source monitoring deficits were higher in FEP individuals compared to those with UHR persistence/transition.

**Conclusion:**

High levels of basic self-disturbance may be a useful predictor marker of poor prognosis in UHR patients.

Disturbance of the “basic self” has been suggested to be a potential core phenotypic marker of schizophrenia spectrum disorders, with implications for etiological research and for early risk identification and intervention.^1–4^ The “basic self” refers to the immediate (“pre-reflective”) awareness of our thoughts, actions, and experiences. The concept includes the sense of agency of action and the sense of ownership of experience (“first-person perspective”),^5^ which are implicit aspects of normal selfhood and facilitate engagement with others and the world.^6^ Instability in the basic self can manifest in various anomalous subjective experiences, which can, over time, intensify and crystallize into positive and negative psychotic symptoms.^7^

The gold standard measure of basic self-disturbance is the interview-based Examination of Anomalous Self-Experience (EASE).^8^ A substantial body of research employing the EASE and pre-EASE scales highlights that basic self-disturbance is a trait vulnerability characteristic of schizophrenia spectrum disorders. It has been identified during the prodromal phase of these disorders^2^ and may serve as a predictor for the onset of schizophrenia spectrum disorders in first-admission patients.^9^ Furthermore, it has been linked to the transition to full-threshold psychosis in individuals at ultra-high risk (UHR) for psychosis.^1,10^

Several proposals have been made regarding the neural underpinnings of basic self-disturbance (see Nelson et al.^11^ and Martin et al.^12^ for a summary). A neurophenomenological model introduced by Nelson et al.^13,14^ proposed that the neurocognitive constructs of source monitoring deficits and aberrant salience—both associated with psychosis risk and prominent in schizophrenia spectrum disorders—may be correlated with basic self-disturbance. Together, these phenomenological and neurocognitive constructs may predict persistence and worsening of psychotic symptoms in high risk individuals. Source monitoring deficits refer to a reduced ability to correctly identify the source or origins of experiences—for example, to know whether a stimulus is generated by oneself or another person or whether it is real or imagined. Aberrant salience refers to difficulty suppressing attention to familiar or unimportant information, resulting in an excessive attention to irrelevant stimuli. These neurocognitive disturbances might contribute to the subjective (phenomenological) disturbances associated with basic self-disturbance, such as hyper-reflexivity, confusion of self-other boundaries, and diminished “ownership” of mental content.^15^

A preliminary test of the cross-sectional aspect of this model was conducted via assessment of UHR individuals, individuals with first-episode psychosis (FEP), and healthy controls using a variety of clinical measures, including the EASE, and neurocognitive and neurophysiological measures of source monitoring deficits and aberrant salience. The findings provided partial support for the neurophenomenological model. Specifically, source monitoring deficits were found to explain a substantial amount (39.8%) of the variance in EASE scores. However, no relationship was found with aberrant salience, which instead correlated with general psychopathology, particularly with positive psychotic symptoms.^11,16^

In order to examine whether these clinical and neuro-measures are associated with the longitudinal persistence and worsening of attenuated psychotic symptoms in those at high risk, the UHR sample was reassessed 12 months later. The current paper reports on these longitudinal data. The aim was to compare baseline basic self-disturbance and neuro-measures of source monitoring and aberrant salience in UHR individuals who had persistent UHR status (i.e., non-remission) or who transitioned to threshold psychotic disorder (referred to as the UHR persistence/transition group) with UHR individuals who remitted from UHR status (UHR-remission group) and with FEP individuals.

## Methods

### Participants

The sample consisted of 43 UHR individuals and 38 individuals with FEP. Clinical outcome status of the UHR group was determined at 12-month follow-up using the Comprehensive Assessment of At-Risk Mental States (CAARMS)^17,18^ or by consulting state medical records. Participants were categorized as belonging to the UHR persistence/transition group (*n* = 25, consisting of 21 non-remitted UHR cases and 4 cases who transitioned to psychosis) and the UHR remitted group (*n* = 18). The assessment of the FEP group (*n* = 38) was based exclusively on the data collected at baseline. The study was approved by the local research and ethics committee, and participants provided written informed consent.

### Measures

Clinical measures included the EASE (total score and five subscale scores: cognition and stream of consciousness; self-awareness and presence; bodily experience; demarcation/transitivism; and existential reorientation),^8^ the CAARMS positive symptoms scale,^17,18^ the Brief Psychiatric Rating Scale (BPRS),^19^ the Scale for the Assessment of Negative Symptoms (SANS),^20^ and the Social and Occupational Functioning Assessment Scale (SOFAS).^21^ As per previous analyses of the neuro-measures in this sample, composite scores across measures of aberrant salience and source monitoring were used. These composite scores were derived by summing the unit-weighted z-scores of the constituent neurocognitive and neurophysiological (EEG) tests for aberrant salience and source monitoring deficits, respectively.^11,16^

### Data analysis

Analyses were conducted using R version 4.1.2. Baseline clinical and neuro-measures were compared between groups using analysis of variance (ANOVA). Results were corrected for multiple comparisons using the false discovery rate (FDR).^22^ For measures surviving FDR correction, post hoc pairwise comparisons were conducted using Tukey t-tests. Additionally, for exploratory purposes, a logistic regression was performed to determine whether baseline clinical measures predicted remission versus persistence/transition status. Neurocognitive measures were not included due to high rates of missingness.

## Results

**Figure 1.**
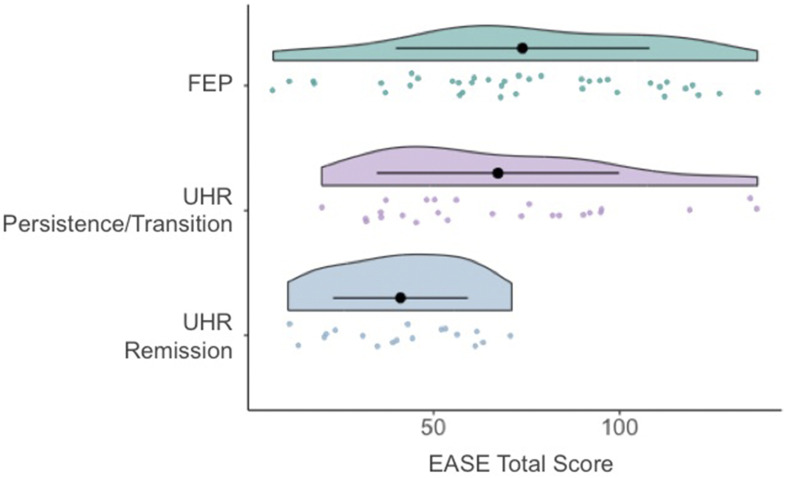
Baseline EASE total scores in the two UHR outcome groups and the FEP group.

Table 1 presents the baseline data of the UHR outcome groups and the FEP group, while Table 2 summarizes baseline differences between groups for the variables that demonstrated significant differences. Baseline EASE scores (total score) were significantly higher in the UHR persistence/transition group compared to the UHR-remission group (*p* = 0.02), with no differences observed between the UHR persistence/transition and FEP groups (see Table 2; Figure 1). Specifically, the subdomains of self-awareness and presence, bodily experience, and demarcation/transitivism were significantly higher in the UHR persistence/transition group compared to the UHR-remission group. Both UHR groups had lower baseline CAARMS positive symptoms compared to the FEP group, but these UHR outcome groups did not differ from each other. FEP individuals had greater source monitoring deficits compared to the UHR persistence/transition group. No significant between-group differences were observed for baseline BPRS, SOFAS, SANS, or aberrant salience scores.

**Table 1.** Baseline data of the UHR outcome groups and the FEP group

Variable	UHR remission			UHR Persistence/Transition			FEP		
	N	Mean	SD	N	Mean	SD	N	Mean	SD
EASE total	18	41.2	18.1	25	67.4	32.5	38	73.9	34.0
Cognition and stream of consciousness	18	23.7	9.2	25	31.1	14.0	38	33.1	14.2
Self-awareness and presence	18	12.6	7.3	25	23.9	13.7	38	23.3	13.5
Bodily experience	18	2.2	2.0	25	7.7	7.5	38	8.1	6.6
Demarcation / Transitivism	18	0.4	1.2	25	2.6	3.4	38	2.0	2.8
Existential reorientation	18	2.3	4.5	25	4.6	6.5	38	7.4	5.3
CAARMS positive symptoms	18	40.2	14.5	25	44.8	15.1	37	72.0	16.1
BPRS total	18	48.1	8.5	25	50.9	8.9	37	51.8	13.5
SANS total	18	28.9	21.2	25	27.4	14.9	37	22.4	16.5
SOFAS	18	52.4	8.7	25	53.0	8.2	37	52.3	11.6
Source monitoring	12	0.2	1.9	15	−0.9	2.1	18	2.0	2.8
Aberrant salience	12	−0.9	3.1	16	0.3	1.7	20	−0.3	4.1

UHR = Ultra-High Risk for Psychosis; FEP = First-Episode Psychosis; EASE = Examination of Anomalous Self-Experience; CAARMS = Comprehensive Assessment of At-Risk Mental States; BPRS = Brief Psychiatric Rating Scale; SANS = Scale for the Assessment of Negative Symptoms; SOFAS = Social and Occupational Functioning Scale.

**Table 2.** Baseline differences between the UHR outcome groups and the FEP group

Variable	F	*p*	eta Sq	Post hoc pairwise comparisons					
				UHR-R vs UHR-P/T		UHR-R vs FEP		UHR-P/T vs FEP	
				t value	*p*	t value	*p*	t value	*p*
EASE total	7.07	0.002^*^	0.15	2.75	0.020^*^	3.72	0.001^*^	0.83	0.685
- Cognition and stream of consciousness	3.16	0.048	0.07	1.81	0.173	2.49	0.038^*^	0.60	0.817
- Self-awareness and presence	5.46	0.006^*^	0.12	2.95	0.011^*^	3.01	0.010^*^	−0.20	0.979
- Bodily experience	6.13	0.003^*^	0.14	2.89	0.014^*^	3.35	0.003^*^	0.26	0.964
- Demarcation/Transitivism	3.22	0.045	0.08	2.49	0.039^*^	1.94	0.132	−0.83	0.685
- Existential reorientation	5.60	0.005^*^	0.13	1.33	0.381	3.22	0.005^*^	1.98	0.122
CAARMS positive symptoms	35.93	<0.001^*^	0.48	0.97	0.596	7.18	<0.001^*^	6.81	<0.001^*^
BPRS total	0.68	0.508	-	-	-	-	-	-	-
SANS total	1.11	0.334	-	-	-	-	-	-	-
SOFAS	0.04	0.960	-	-	-	-	-	-	-
Source monitoring	6.11	0.005^*^	0.23	−1.21	0.453	1.98	0.130	3.45	0.004^*^
Aberrant salience	0.47	0.626	-	-	-	-	-	-	-

UHR-R = Ultra-High Risk for Psychosis Remission Group; UHR-P/T = Ultra-High Risk for Psychosis Persistence/Transition Group; FEP = First-Episode Psychosis; EASE = Examination of Anomalous Self-Experience; CAARMS = Comprehensive Assessment of At-Risk Mental States.

^*^FDR-corrected *p* < .05.

An exploratory logistic regression indicated that baseline EASE total score predicted UHR clinical outcome group status (remission vs. persistence/transition; see Table 3). Other clinical scales, including BPRS, CAARMS positive symptoms, SANS, and SOFAS were not significant predictors of UHR outcome status.

**Table 3.** Baseline prediction of UHR outcome status (logistic regression)

	Estimate	Std. Error	z value	Pr(>|z|)
EASE total	0.05	0.02	2.38	0.018^*^
SOFAS	<0.01	0.06	−0.02	0.983
CAARMS positive symptoms	−0.02	0.03	−0.68	0.496
BPRS total	0.01	0.06	0.16	0.874
SANS total	−0.03	0.03	−0.97	0.332

EASE = Examination of Anomalous Self-Experience; SOFAS = Social and Occupational Functioning Scale; CAARMS = Comprehensive Assessment of At-Risk Mental States; BPRS = Brief Psychiatric Rating Scale; SANS = Scale for the Assessment of Negative Symptoms.

^*^p < .05.

## Discussion

The findings suggest that high levels of basic self-disturbance at baseline (as indicated by EASE total score) are associated with persistence of UHR status and transition to psychosis in UHR individuals. These results are consistent with previous findings linking basic self-disturbance with transition to psychosis in this population.^10^ Furthermore, while the UHR persistence/transition group had lower CAARMS positive symptoms scores than the FEP group at baseline, their EASE scores did not differ at baseline from the FEP group. This suggests that basic self-disturbance may be a more useful trait marker of risk for attenuated psychotic symptoms being persistent or intensifying over time than severity of positive symptoms. The latter may fluctuate to a larger extent than self-disturbance, a proposal that could be investigated using ecological momentary assessment (EMA), as in a recent study by Baklund and colleagues.^23^

No association was found between UHR clinical outcome and the neuro-measures of source monitoring deficits and aberrant salience. This is not consistent with the neurophenomenological model proposed by Nelson et al.,^13,14^ which posited that higher levels of source monitoring deficits and aberrant salience would be expected in the UHR persistence/transition group, compared to the remission group. It is possible that the small sample size, the low number of transitioned cases, the phenotypic heterogeneity of the combined persistence/transition group, the substantial amount of missing data on these measures, and the short follow-up period may have contributed to the absence of association between these variables. A larger scale study, with a wider battery of measures and the inclusion of a clinical comparison group, is currently underway to test this model more comprehensively.^24^

## Conclusion

The current study indicates that basic self-disturbance is associated with worse clinical outcomes in UHR individuals, namely, persistence of UHR status or transition to psychosis. These findings underline the possible utility of measuring self-disturbance for identifying the subgroup of UHR patients with a poorer prognosis and for guiding etiological and treatment research. Further investigation of neurocognitive and neural underpinnings of self-disturbance is underway.
